# KlebSeq, a Diagnostic Tool for Surveillance, Detection, and Monitoring of Klebsiella pneumoniae

**DOI:** 10.1128/JCM.00927-16

**Published:** 2016-09-23

**Authors:** Jolene R. Bowers, Darrin Lemmer, Jason W. Sahl, Talima Pearson, Elizabeth M. Driebe, Bette Wojack, Michael A. Saubolle, David M. Engelthaler, Paul Keim

**Affiliations:** a Translational Genomics Research Institute, Flagstaff, Arizona, USA; b Microbial Genetics and Genomics, Flagstaff, Arizona, USA; c Banner Health, Phoenix, Arizona, USA; Medical College of Wisconsin

## Abstract

Health care-acquired infections (HAIs) kill tens of thousands of people each year and add significantly to health care costs. Multidrug-resistant and epidemic strains are a large proportion of HAI agents, and multidrug-resistant strains of Klebsiella pneumoniae, a leading HAI agent, have caused an urgent public health crisis. In the health care environment, patient colonization by K. pneumoniae precedes infection, and transmission via colonization leads to outbreaks. Periodic patient screening for K. pneumoniae colonization has the potential to curb the number of HAIs. In this report, we describe the design and validation of KlebSeq, a highly informative screening tool that detects Klebsiella species and identifies clinically important strains and characteristics by using highly multiplexed amplicon sequencing without a live-culturing step. We demonstrate the utility of this tool on several complex specimen types, including urine, wound swabs and tissue, and several types of respiratory and fecal specimens, showing K. pneumoniae species and clonal group identification and antimicrobial resistance and virulence profiling, including capsule typing. Use of this amplicon sequencing tool to screen patients for Klebsiella carriage could inform health care staff of the risk of infection and outbreak potential. KlebSeq also serves as a model for next-generation molecular tools for public health and health care, as expansion of this tool can be used for several other HAI agents or applications.

## INTRODUCTION

Klebsiella pneumoniae has been a leading health care-acquired infection (HAI) agent for decades (1, 2). The emergence of multidrug-resistant K. pneumoniae, especially extended-spectrum β-lactamase (ESBL) producers and carbapenemase producers, has elevated the morbidity and mortality rates and health care costs associated with K. pneumoniae to highly significant levels (3–6). Health care- and outbreak-associated strain types of K. pneumoniae that appear highly transmissible and have a propensity for antimicrobial resistance (AMR) or virulence gene acquisition are a growing proportion of the K. pneumoniae species (7–18). Sequence type 258 (ST258), the crux of the worldwide carbapenemase-producing Enterobacteriaceae (CPE) threat, has disseminated rapidly around the world's health care systems despite its recent emergence (17). Its progenitor strains in clonal group 258 (CG258) also cause outbreaks and carry many important ESBL- and carbapenemase-encoding genes (9, 19–21). Several other strain types, such as those in CG14, CG20, and CG37, also frequently appear as multidrug resistant and in outbreak situations (7, 10, 12, 15).

Host colonization is likely an important reservoir driving the transmission of these strains. In the health care environment, intestinal colonization of K. pneumoniae is a risk factor for infection (22–24), and carriers of CPE are at high risk for invasive disease (25). Rates of CPE and ESBL-producing K. pneumoniae colonization are rising in patient and health care worker populations, increasing the size of the reservoir and increasing chances of transmission (26, 27). Asymptomatic transmission of multidrug-resistant strains is rapid (16, 28), and transmission events that lead to outbreaks often go undetected (29, 30). Early detection of K. pneumoniae colonization of patients, especially multidrug-resistant K. pneumoniae or epidemic strain type colonization, is now considered critical to infection control (24, 30–33).

Infection control programs that include the detection and isolation of carriers have repeatedly been successful in markedly decreasing multidrug-resistant or epidemic strain infections (31, 34–37), but this practice is uncommon for several reasons. Many of these programs use culture-based methods for detecting CPE or ESBL producers, which have several limitations, including turnaround time, narrow application, fair sensitivity and specificity, and extensive labor for high-throughput screening (31, 38). PCR-based assays are rapid but often use DNA from culture, and a limited number of tests can be run simultaneously, potentially missing important AMR genes not previously known to circulate in a given locale (31, 39).

Next-generation sequencing has gained a foothold in health care with whole-genome sequencing (WGS) for outbreak detection, transmission mapping, and source tracing (40, 41), microbiome sequencing (e.g., targeted 16S rRNA gene sequencing) to understand microbial population structure (42, 43) and with metagenomic sequencing to attempt to determine all of the genetic factors present (44). Although metagenomic sequencing does not require an *a priori* understanding of the genetic targets in a clinical sample, it does have significant drawbacks, limiting its translation to the clinical microbiology laboratory. Chief among these are that (i) the required amount of sequencing space increases the cost and time, (ii) limited coverage across targets lessens the confidence in diagnostic calls, and (iii) the necessary computing power and highly complex analysis limit the ability for local analysis. Targeted amplicon sequencing, on the other hand, allows for rapid, cost-effective, highly multiplexed, and accurate detection of numerous clinically important targets directly in clinical samples (45). Such assays have recently been approved by the FDA for clinical diagnostics (46).

In this report, we describe a new amplicon sequencing tool, KlebSeq, for screening and surveillance that detects and characterizes Klebsiella bacteria in complex samples such as wound and nasal swabs or fecal samples without culturing. KlebSeq includes a sizeable panel of assays for species identification, strain identification, and important AMR and virulence gene targets designed to generate information for health care epidemiology and infection prevention. KlebSeq also includes an analysis pipeline for instant interpretation of the data. Results from the screening of a patient population with this system would rule in or rule out the possibilities of particular transmission events and identify patients carrying high-risk strains like ST258 or other multidrug-resistant Klebsiella strains. The highly multiplexed nature of KlebSeq greatly expands the capacity of a single sequencing run, minimizing costs, and allows for high-throughput patient sample testing. This innovation can also serve as a foundation system on which to build in other HAI agents such as Escherichia coli or Staphylococcus aureus and their multiple AMR mechanisms or as a model for many other applications.

## MATERIALS AND METHODS

### Samples.

Isolates for target identification and assay validation and DNA extracted from clinical specimens were acquired through collaborations with a large hospital reference laboratory that receives specimens from 10 system-wide medical centers in Arizona and from a high-volume private reference laboratory that receives specimens from regional inpatient, long-term care, and outpatient facilities. Isolates were identified with Vitek 2 (bioMérieux). Clinical specimen types included various respiratory specimens (nasal, ear, and throat swabs; sputum samples; tracheal aspirates; and bronchial alveolar lavage samples), urine, and wound swabs or tissue. DNA was extracted from isolates with the Qiagen DNeasy Blood and Tissue kit with additional lytic enzymes when appropriate. DNA was extracted from clinical specimens by NucliSENS easyMAG (bioMérieux, Durham, NC). DNA from healthy donor fecal samples was acquired from a family microbiome study; samples had been collected from members of seven families over multiple time points. DNA was extracted in accordance with the Earth Microbiome Project protocol (47). All of the samples were obtained from studies approved by the institutional review boards of the participating institutions.

### Assay target identification and assay design.

**FIG 1:**
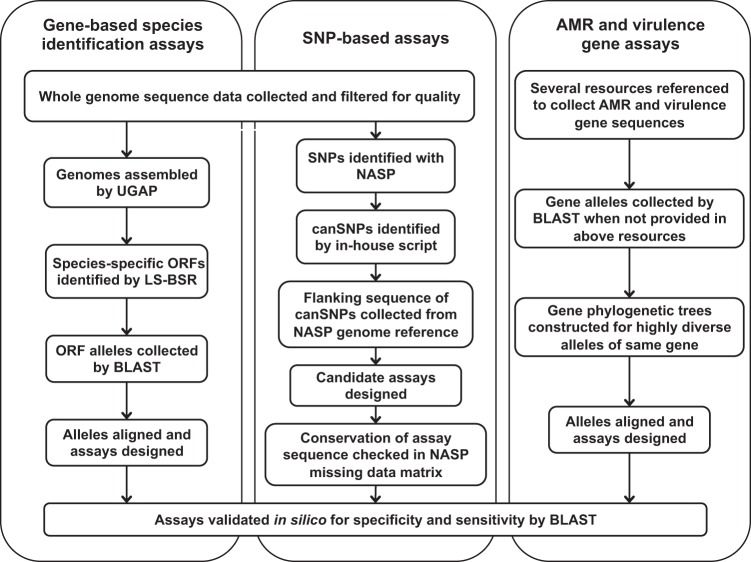
Workflow of the amplicon sequencing target library and assay development pipeline.

Figure 1 illustrates the methodologies and resources, also described below, utilized to amass a target library and develop several types of amplicon sequencing assays.

### WGS, single nucleotide polymorphism (SNP) detection, and phylogenetic analysis.

In-house genome libraries were prepared from 31 Klebsiella isolates and 6 non-Klebsiella isolates (to validate KlebSeq assays) with a 500-bp insert size with the KAPA Library Preparation kit and Standard PCR Library Amplification (Kapa Biosystems, Wilmington, MA) and sequenced on Illumina's GAIIx or MiSeq. Additional in-house genomes that we have previously described were also included and comprised 111 K. pneumoniae, 1 K. quasipneumoniae, and 5 K. variicola genomes. Public genome sequence data from 256 K. pneumoniae, 18 K. quasipneumoniae, and 13 K. variicola isolates were downloaded from the SRA database (http://www.ncbi.nlm.nih.gov/Traces/sra/), and genome sequence data from 177 K. pneumoniae, 4 K. quasipneumoniae, and 11 K. variicola isolates were downloaded from the Assembly database (http://www.ncbi.nlm.nih.gov/assembly), and all passed filters for high quality; i.e., assemblies and SRA data aligned with ≥80% of MGH 78578 or ≥88% of the strict core genome multilocus sequence typing (scgMLST) references (described below), SRA data at a ≥10× read depth. Accession numbers are listed in Tables S1 and S2 in the supplemental material.

NASP (48), developed for microbial genome analysis, was used to detect SNPs among genomes (see Table S1 in the supplemental material). In brief, reads were aligned with a reference genome, either one concatenated from scgMLST alleles (10) or MGH 78578 (GenBank accession no. CP000647) with Novoalign V3.04.04 (Novocraft Technologies, Selangor, Malaysia) and SNPs were called with GATK version 2.7-2 (49). Data filtered out included SNP loci with <10× coverage or with <90% consensus in any one sample, regions duplicated in the reference genome as identified by Nucmer, and SNP loci that were not present in all of the genomes in the data set. In NASP, results were output in a SNP matrix from a core genome common to all of the isolates in the analysis. Phylogenetic trees were generated from the NASP SNP matrices with MEGA 6.0 (50) and subsequently plotted by means of ITOL v2 or v3 (51).

### Genomic target identification.

To find whole gene targets for assay design, selected genomes were assembled with UGAP (https://github.com/jasonsahl/UGAP), which uses the SPAdes genome assembler, version 3.6, for this work (52). Assemblies were then run through LS-BSR (53), which generates a list of open reading frames (ORFs) that have high identity among target species genomes and that have low identity or are not present in nontarget genomes. Alleles of the candidate target ORFs were collected by BLAST, including alleles from nontarget genomes, if present. Lastly, alleles of candidate ORFs were aligned for assay design. Canonical SNPs (canSNPs) were identified from the SNP matrix generated by NASP. Sequence flanking each SNP was collected from the NASP reference genome.

### AMR and virulence gene target collection.

AMR and virulence gene sequences were identified and collected in several ways, including from http://www.lahey.org/studies/other.asp#table1, http://www.lahey.org/qnrstudies/, the Klebsiella BIGSdb at http://bigsdb.web.pasteur.fr/klebsiella/, public literature, and the NCBI at http://www.ncbi.nlm.nih.gov. Public literature included a paper by Holt et al. (54) in which a species-wide analysis of K. pneumoniae genomes revealed several siderophore systems and other virulence factors associated more with infectious than with colonizing strains. AMR genes included the major ESBL and carbapenemase genes and plasmid-mediated quinolone resistance determinants, as well as the *gyrA* and *parC* chromosomal genes, several aminoglycoside resistance genes, trimethoprim-sulfamethoxazole, tetracycline, streptomycin, chloramphenicol, and fosfomycin resistance genes, and the recently discovered plasmid-mediated colistin resistance gene *mcr-1*. Virulence targets included several siderophore systems, for which multiple genes from each were used as assay targets; the regulator of the mucoid phenotype (an indicator of hypervirulence); the *wzi* gene for capsule typing, for which we used the previously published assay (55); and two genes highly associated with invasive infection, pK2044_00025 and pK2044_00325 (54). For genes that consist of highly diverse alleles, for example, *bla*_CTX-M_, *qnrB*, or *dfrA*, phylogenetic trees based on nucleotide sequences were generated in order to group similar alleles for assay design.

### Assay design and validation.

Gene-based target alleles were aligned in SeqMan (DNAStar, Madison, WI) to identify conserved regions for primer design, and assays were designed with guidance from RealTimeDesign (Biosearch Technologies, Petaluma, CA), or gene-based assays were generated with AlleleID (Premier Biosoft, Palo Alto, CA), which designs assays to capture alleles in an alignment rather than individual sequences. SNP assay primers were designed with RealTimeDesign, and primer sequences were checked for conservation in the NASP SNP matrix. Lastly, assays were run through BLAST (http://blast.ncbi.nlm.nih.gov/Blast.cgi) to check for cross-reactivity with other relevant targets or species, including human. Universal tails were added to each primer sequence for library preparation as described below. The assays and their primer sequence are listed in Table S3 in the supplemental material.

Individual assays were screened across positive controls when they were accessible and screened across several isolate genomic DNAs (gDNAs) to test robustness. Additionally, multiplex PCR was validated by initial gene-specific PCR (described below), followed by PCR product dilution and then screening of individual assays by Sybr green-based quantitative PCR (qPCR). For this, 10-μl reaction mixtures of 1× Platinum SYBR green qPCR SuperMix (ThermoFisher Scientific, Waltham, MA), 200 nM forward and reverse primers of one assay, and 1 μl of diluted multiplex PCR product were run at 95°C for initial denaturation for 4 min and then 40 cycles of 95°C for 15 s and 60°C for 1 min. Lastly, several panels of known isolate DNAs were screened by the amplicon sequencing method to test the sensitivity and specificity of the species and strain identification assays. AMR and virulence gene assays were validated by comparing amplicon sequencing results with WGS data.

### Amplicon library preparation and sequencing.

Amplicon library preparation with universal tails was described in detail previously (56). Here, assays were combined into three assay pools for multiplex PCR (see Table S3 in the supplemental material), requiring three initial PCRs for each sample. The initial gene-specific PCR mixture comprised 12.5 μl of Kapa Multiplex PCR Mastermix (Kapa Biosystems, Wilmington, MA), 10 μl of primer mix (final concentration of 200 nM each), and 2.5 μl of template DNA from each sample and was denatured at 95°C for 3 min; cycled 25 times at 95°C for 15 s, 60°C for 30 s, and 72°C for 1 min 30 s; and subjected to a final extension at 72°C for 1 min. The three multiplex PCR products from the same sample were combined, and PCR products were cleaned with 1× Agencourt AMPure XP beads (Beckman Coulter, Indianapolis, IN). A second PCR with the universal tail-specific primers added Illumina's sample-specific index and sequencing adapters. This PCR mixture comprised 12.5 μl of 2× Kapa HiFi HotStart Ready Mix (Kapa Biosystems), 400 nM each primer, and 1 to 10 μl of cleaned gene-specific PCR product and was denatured at 98°C for 2 min; cycled 6 to 12 times at 98°C for 30 s, 65°C for 20s, and 72°C for 30 s; and subjected to a final extension at 72°C for 30 s. Final PCR products were cleaned with 0.8× Agencourt AMPure XP beads (Beckman Coulter). Amplicon libraries from individual samples were quantified by qPCR with the Kapa Library Quantification kit (Kapa Biosystems). Samples were then pooled in equimolar concentrations for sequencing on the Illumina MiSeq platform with the 2x250bp version 2 kit.

### Analysis.

**FIG 2:**
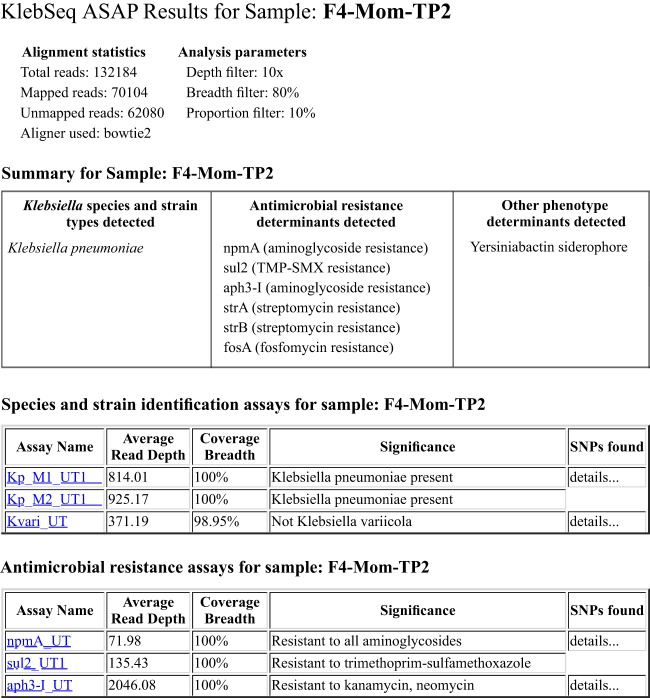
Partial sample output of KlebSeq ASAP report; some AMR gene assays and the virulence gene assays are hidden from view to limit the size of the image. The top box shows a summary of what was detected according to selected ASAP filters. Details of each assay appear below that. If additional SNPs are detected in comparison to the assay reference, hovering over “details…” expands a list of the SNPs. Clicking on an assay name pops up a graph of coverage depth across the reference sequence.

Amplicon sequencing results were automatically analyzed with a newly developed amplicon sequencing analysis pipeline (ASAP) (45) that uses a JavaScript Object Notation (JSON) file customized to describe all of the assays in a multiplex. The information in the JSON file includes (i) a category for each assay (presence/absence, SNP, gene variant, or region of interest) that dictates how ASAP will report results and (ii) reference sequences for read mapping. In ASAP, amplicon sequence reads are first trimmed of adapter or readthrough sequences with Trimmomatic (57) and then mapped to the reference sequences with an aligner of choice. BAM alignment files are analyzed alongside the JSON file assay descriptions to determine the presence, percent identity, and breadth and depth of coverage of the reference and proportions of nucleotide polymorphisms for each amplicon. User-defined parameters for KlebSeq-prepared samples included the bowtie2 aligner (58) for all of the assays except for *wzi*, for which bwa (59) was chosen (because the reference sequence is shorter than the expected amplicon and reads need to be clipped to align [55]), and thresholds for determining results of screening included percent identities listed in Table S3 in the supplemental material, 80% breadth at 100× depth of coverage for isolate DNA, 80% at 20× (clinical specimens) or 10× (fecal specimens) depth, and a ≥10% proportion of polymorphism for informative SNP loci for complex-specimen DNA (meaning that at least 10% of the reads had to share a SNP state at a given locus for it to be reported). For WGS-prepared data, the parameters were bwa aligner (for clipping) and 80% breadth at 5× depth. The ASAP output includes an XML file containing details of the analysis of each assay target for each sample, which can be converted into a webpage interface by XSLT transformations. An example of a KlebSeq ASAP output for one sample is shown in Fig. 2. SeqMan NGEN (DNAStar, Madison, WI) and Tablet (60) were used to verify results.

### KlebSeq validation.

**FIG 3:**
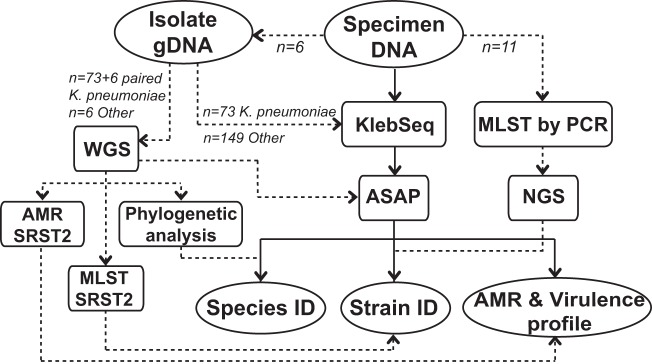
Workflow of the validation of KlebSeq. Dotted lines are methods used to confirm results from the workflow in solid lines (KlebSeq of specimen DNA). Strain identification validation was performed for 73 isolates plus 6 isolates that were cultured from KlebSeq-tested specimens (Tables 2 and 3). MLST PCR and sequencing were performed with 11 specimen DNA samples (Table 3). AMR gene detection validation is described in the text. The overall specimen KlebSeq results are in Table S4 in the supplemental material. NGS, next-generation sequencing.

Figure 3 and the following text outline the processes used to validate KlebSeq, and Table S3 in the supplemental material shows the extent to which each assay was validated in multiplex. First, WGS data from 73 K. pneumoniae samples were analyzed for AMR genes, subjected to MLST via SRST2 (61) and species identification confirmation via phylogenetic analysis, and also analyzed by ASAP. gDNA from these same 73 samples plus gDNA from 149 other species was screened with KlebSeq. To validate KlebSeq's K. pneumoniae strain identification and AMR gene profiles in specimens, six isolates that had been cultured and identified in six of the specimens were sequenced and analyzed. Additionally, a PCR for MLST was performed with selected specimen DNAs by the protocol from the Klebsiella BIGSdb. DNA libraries from the PCR products were prepared for sequencing by the same protocol as for whole gDNA. Sequence data were run through SRST2 to determine the ST of the K. pneumoniae present in the specimen.

## RESULTS

### Phylogenetic analysis and canSNP identification.

With the Klebsiella scgMLST (10) assembly as a reference, SNPs among a diverse set of genomes from K. pneumoniae and genomes from newly defined K. quasipneumoniae (22 from the public databases and 1 from in-house isolates) and K. variicola (24 from the public databases and 5 from in-house isolates) were identified with NASP. canSNPs that differentiate K. quasipneumoniae and K. variicola from K. pneumoniae were selected for assay development.

**FIG 4:**
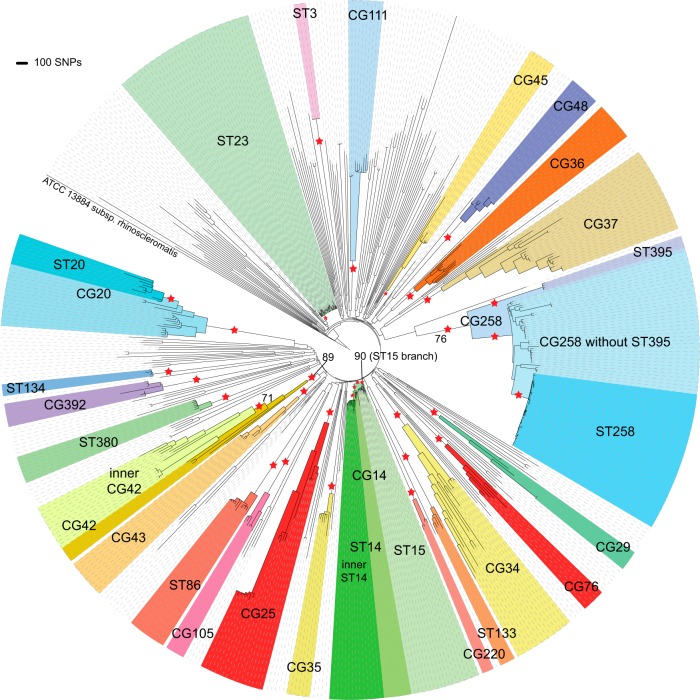
Maximum-parsimony tree with 100 bootstraps of the SNPs among 548 K. pneumoniae genomes. Major clonal groups are colored, and locations of canSNPs for strain identification assays are marked with stars. All of the branches labeled with canSNPs had >99% bootstrap support, except for the three branches indicated.

With the reference genome MGH 78578 and 547 diverse K. pneumoniae genomes, NASP generated a SNP matrix from which canSNPs for each of the major clonal groups were selected for assay development. Clonal groups and locations of canSNPs identifying 21 clonal groups and 12 STs in the context of the K. pneumoniae species are illustrated in Fig. 4. Redundancy was intentionally included in identifying canSNPs for the most epidemic strains of K. pneumoniae, such as ST14, ST20, and strains in CG258, in order to increase sensitivity and confidence in positive results.

### Assay development.

The identification of genomic targets, canSNPs, and AMR and virulence genes and subsequent assay design resulted in two assays specific to K. pneumoniae (Kp-M1 and Kp-M2), one each for K. oxytoca (Koxy_UT), K. variicola (Kvari_UT), and K. quasipneumoniae (Kquasi_UT), 37 assays to identify clonal groups or lineages within clonal groups of K. pneumoniae, 76 AMR gene assays, and 15 virulence gene assays (see Table S3 in the supplemental material). The canSNP states for each strain identification assay are specific to that clonal group of K. pneumoniae, except in the case of CG35, where the amplicon must match the reference sequence 98%, allowing up to four additional SNPs, in order to be called CG35. Otherwise, identity thresholds for each strain identification assay are optional; they merely make the assays completely Klebsiella specific, regardless of the canSNP state.

### KlebSeq validation on isolate DNA.

**FIG 5:**
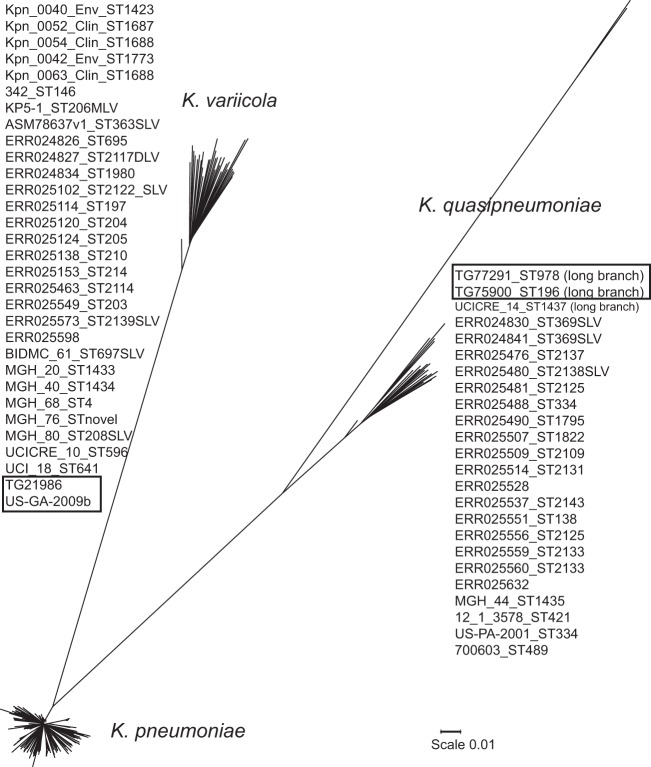
Neighbor-joining tree with 100 bootstraps of the SNPs in the diverse set of K. pneumoniae, K. variicola, and K. quasipneumoniae genomes used in this study. Unknown isolates that were identified as K. variicola and K. quasipneumoniae are boxed.

To validate the species and clonal group identification assays, gDNA from 73 K. pneumoniae isolates whose whole genomes were sequenced (4 of which were later identified as K. quasipneumoniae and K. variicola [see below]), 22 K. oxytoca isolates, and 157 other enteric opportunistic pathogen isolates, which included E. coli, Enterobacter aerogenes, E. amnigenus, E. cloacae, E. hormaechei, Enterococcus faecalis, E. faecium, an unknown Enterococcus species, Proteus mirabilis, Providencia stuartii, and Serratia marcescens, and 1 Acinetobacter baumannii isolate, were screened with KlebSeq. Sensitivity and specificity results of the species identification assays compared with clinical microbiological identification (Vitek 2) are in Table 1. With the redundancy built into the multiplex by including two assays, Kp-M1 and Kp-M2, that target two different K. pneumoniae species-specific genes (M1 and M2), 100% sensitivity is achieved. One isolate previously identified as K. pneumoniae was identified as K. quasipneumoniae, and two were identified as K. variicola. These isolates' whole genomes were added to the phylogenetic analysis of these three species that was previously run to find the species-specific canSNPs (see Materials and Methods). The K. quasipneumoniae and K. variicola genomes identified by our assay clustered with their respective species in the phylogeny (Fig. 5). Clinical methods do not currently distinguish among all three of these species, so assay sensitivity and specificity were not calculated for K. quasipneumoniae and K. variicola (Table 1).

**TABLE 1:** Results of KlebSeq species identification assays of genomic DNA from isolates whose whole genomes were also sequenced, DNA from specimens for which clinical culture results are known, and DNA from specimens with unknown content

DNA type (no. of samples) and species identified by clinical methods or parameter^*a*^	Total no. screened	No. of isolates identified by amplicon sequencing assay^*b*^					
		Kp-M1	Kp-M2	Kp-M1 + Kp-M2	Kquasi_UT	Kvari_UT	Koxy_UT
Isolate DNA (252)							
K. pneumoniae	69	68	67	69	0	0	0
K. quasipneumoniae	2	0	0	0	2	0	0
K. variicola	2	2	0	2	0	2	0
K. oxytoca	14	0	0	0	0	0	14
Nontarget species	149	2/88	0/88	2/88	0/155	0/155	0/135
% Sensitivity		99	97	100	100	100	100
% Specificity		98	100	98	100	100	100
Urine DNA (46)							
K. pneumoniae	16	14	15	16	2 (1 mix)^*c*^	1 (mix)^*c*^	0
K. oxytoca	6	1	1	1 (CG34)	0	0	6
Other species	24	1	1	1	0	0	0
Unknown	0	0	0	0	0	0	0
% Sensitivity		88	94	100			100
% Specificity		90	93	93			100
Wound DNA (40)							
K. pneumoniae	1	0	0	0	0	1	0
K. oxytoca	1	0	0	0	0	0	1
Other species	31	0	0	0	0	0	0
Unknown	7	1	1	1 (CG29)	0	0	1
% Sensitivity		0	0	0			100
% Specificity		100	100	100			100
Respiratory specimen DNA (87)							
K. pneumoniae	6	6	6	6	0	0	0
K. oxytoca	1	0	0	0	0	0	1
Other species	77	7 (1 ST258)	5	7 (2 CG36, 1 CG37)	0	0	1
Unknown	3	0	0	0	0	0	0
% Sensitivity		100	100	100			100
% Specificity		91	94	91			99
Fecal specimen DNA (89)	89	9	5	9	1 (mix)^*c*^	3 (2 mix)^*c*^	13
All specimens (isolates not included) (262)							
K. pneumoniae	23	20	21	22	2	2	0
K. oxytoca	8	1	1	1	0	0	8
Other species	132	8	6	8	0	0	1
Unknown	99	10	6	10	1	3	14
% Sensitivity		87	91	96			100
% Specificity		94	95	94			99

a K. quasipneumoniae is not distinguished from K. pneumoniae by the clinical identification method used (Vitek 2).

b Kp-M1 and Kp-M2 are K. pneumoniae species identification assays that detect targets M1 and M2 in the K. pneumoniae genome. Kquasi_UT, Kvari_UT, and Koxy_UT are K. quasipneumoniae-, K. variicola-, and K. oxytoca-specific assays, respectively.

c These species were found as mixtures with K. pneumoniae on the basis of a proportion (≥10%) of the sequencing reads containing the species-defining SNP.

Table 2 shows the KlebSeq results of the K. pneumoniae clonal group identification and capsule typing assays of isolate DNA. Each isolate's strain type was correctly captured by the appropriate assays or not captured in cases where no assay was designed for that clonal group. Included in Table 2 are results from partial sequencing of the *wzi* gene for capsule typing. This gave surprisingly clear results, given that approximately 75 bp of the informative region are missing from our sequence output, as the PCR amplicon is approximately 580 bp (55), which is too long to cover with the Illumina version 2 sequencing chemistry. However, full capsule typing by *wzi* sequencing would be possible with longer-read chemistry (i.e., Illumina version 3 chemistry, for 600-bp reads). Results from screening of nontarget organisms showed that several of the K. pneumoniae clonal group assays amplified DNA from other organisms, as expected. An identity threshold can be applied (see Table S3 in the supplemental material); however, all of the SNP states that define a particular clonal group are specific to that clonal group, except for CG35, so the identity threshold is optional except for this assay. Sequence analysis by ASAP reports when a clonal group is present only if the defining canSNP state is present and reports nothing if it is not.

**TABLE 2:** Isolates used for assay validation and results of strain typing by amplicon sequencing^*a*^

Isolate ST	No. of isolates	ASAP strain typing assay result(s)	Capsule typing result(s) by partial *wzi* sequencing^*b*^
ST11	3	CG258, CG258 without 395	wzi-39 or -75, wzi-74, not typeable
ST14	5	CG14, ST14, inner ST14	All wzi-2
ST14 SLV^*d*^	1	CG14	wzi-16
ST15	2	CG14, ST15	All wzi-24 or -45
ST20	2	CG20, ST20	wzi-84, wzi-118
ST23	8	ST23	wzi-1
ST34, ST34 SLV	2	CG34	wzi-114, wzi-12
ST36	2	CG36	All wzi-27 or -79
ST37	2	CG37	wzi-50, wzi-39 or -75
ST39	1	No group	wzi-2
ST42	2	CG42, inner CG42	All wzi-29
ST43	1	CG43	wzi-30
ST45	1	CG45	wzi-133
ST65	1	CG25	wzi-72
ST101	2	CG43	wzi-29, wzi-137
ST107	1	No group	wzi-74
ST111	1	CG111	wzi-63
ST147	1	CG392	wzi-64
ST152	1	CG105	wzi-150
ST228	1	CG34	wzi-116^*c*^
ST234	1	No group	wzi-114
ST249	2	No group	All wzi-128
ST258, no clade	6	CG258, CG258 without ST395, ST258	All wzi-154
ST258, clade 1	3	CG258, CG258 without ST395, ST258, clade 1	All wzi-29
ST258, clade 2	2	CG258, CG258 without ST395, ST258, clade 2	All wzi-154
ST277	1	No group	wzi-97 or -185
ST334	1	K. quasipneumoniae	wzi-68
ST340	2	CG258, CG258 without ST395, ST340	wzi-50, wzi-173
ST376	1	CG42, inner CG42	wzi-2
ST380	1	ST380	wzi-203
ST437	1	CG258, CG258 without 395, ST437	wzi-109
ST636	1	No group	wzi-155
ST719	1	No group	wzi-192
ST776	1	No group	wzi-39^*c*^ or -75^*c*^ or -193^*c*^
ST833	1	CG258, CG258 without 395	wzi-50
ST978	1	K. quasipneumoniae	wzi-212^*c*^
ST1401	1	No group	wzi-96
ST82	2	No group	All wzi-128
ST260	1	No group	wzi-1
ST360 SLV	1	K. variicola	wzi-53
ST427 SLV	1	No group	wzi-64
ST513 SLV	1	No group	wzi-87
ST815 SLV	1	No group	wzi-114^*c*^
ST244 SLV	1	No group	wzi-162^*c*^
ST2006	1	K. variicola	wzi-227
ST2055	1	No group	wzi-14

a Table S2 in the supplemental material lists the genome accession numbers of the isolates.

b The Illumina version 2 chemistry used provides approximately 500 bp of sequence data. The amplicon size for the *wzi* assay is approximately 580 bp (55).

c The *wzi* allele represents the best match; one or more SNPs were present.

d SLV, single-locus variant.

AMR gene detection by amplicon sequencing was validated by comparing ASAP results with AMR gene screening of WGS with SRST2 (61) and with ASAP. Results showed an almost perfect correlation between KlebSeq ASAP and WGS ASAP, indicating that the KlebSeq PCRs are performing well. There were a few discrepancies with SRST2, which reported uncertainty (SNPs or low-coverage indicators) for most of the discrepancies. Some discrepancies were in the presence of the *dfrA* gene. This group of genes is very diverse, so it may be that KlebSeq does not capture the full repertoire of *dfrA* genes. Virulence gene detection was validated by comparing ASAP results from WGS data with those from amplicon sequence data, and results showed concordance. In addition, by targeting multiple genes that are part of the same virulence factors (i.e., siderophore systems), sensitivity and confidence in results were increased.

These results also confirm that KlebSeq is applicable to pure isolates as well as complex specimens. Screening of isolate DNA has the added benefit of traceability of the AMR and virulence genes, which are often carried on mobile genetic elements, to their host. Isolate screening could be used for surveillance and other purposes for identifying or characterizing Klebsiella.

### Specimen sample results.

KlebSeq was run on DNA from 87 respiratory specimens, 46 urine specimens, 40 wound specimens, and 89 fecal samples from healthy individuals (see Table S4 in the supplemental material). Sensitivity and specificity results of the species identification assays compared with those of clinical microbiological methods are shown in Table 1. In most cases, sensitivity was high, except in the wound specimens, where the one sample clinically identified as K. pneumoniae was identified as K. variicola. Some sensitivity and specificity calculations may be misleadingly low, as amplicon sequencing identified some samples as containing K. pneumoniae that actually contained K. quasipneumoniae or K. variicola, and several as containing Klebsiella that went undetected by clinical microbiological methods, including several in which clonal groups were also detected. In the healthy donor specimens, K. oxytoca (*n* = 13) was more prevalent than K. pneumoniae (*n* = 9). Sequencing read depth was low in some samples (see Table S4 in the supplemental material); this may have been due to dilution of the DNA samples before screening, as each was diluted 1:10 in water.

Important Klebsiella clonal groups were detected in multiple specimens (see Table S4 in the supplemental material). In the 17 urine samples positive for K. pneumoniae, clonal identifications included CG34, ST20, CG45, CG392 (which includes the NDM producer ST147 [62], though this sample was negative for *bla*_NDM_), ST133, and CG111. In wounds, the only sample K. pneumoniae positive by KlebSeq was CG29. From respiratory specimens, groups CG37 (*n* = 2), ST134 (*n* = 1), ST258 (*n* = 2), CG36 (*n* = 3), and inner ST14 (*n* = 1) were identified. Interestingly, several clonal groups were identified in the healthy donor fecal specimens as well. In the nine K. pneumoniae-positive samples, the groups included ST20, CG37, and CG76, which are all members of multidrug-resistant outbreak strain types (11, 12, 15), along with ST133 and ST380. ST380 is associated with a K2 capsule type and hypervirulence and causes pyogenic liver abscesses in healthy people, especially those of Asian ethnicity (63). Many Asians are colonized by hypervirulent K1 or K2 capsule strain types; however, the level of risk of subsequent liver infection is unknown (63). For this sample, no *wzi* gene sequence was obtained; thus, the capsule type is unknown. A majority of the K. pneumoniae isolates in our samples did not fall into the major clonal groups targeted by KlebSeq. These strains probably all belong to lesser-known clonal groups, as more studies are showing that many K. pneumoniae infections are caused by nonepidemic, sporadic strains (64, 65).

Numerous and variable AMR genes were detected in the specimens, including different variants of the same gene that confer different phenotypes (see Table S4 in the supplemental material). With sequence-based information, we demonstrate that seven of the K. pneumoniae had key mutations in the *gyrA* gene known to confer resistance to fluoroquinolones. Additionally, several samples contained the *aac*(*6*′)-*Ib* gene for aminoglycoside resistance, and five of those contained the sequence variant *aac*(*6*′)-*Ib-cr* for fluoroquinolone resistance; mixtures of these two genes were also detected. Many of the infection specimens (nonhealthy donor specimens), both positive and negative for K. pneumoniae, were positive for other aminoglycoside resistance genes, as well as tetracycline, trimethoprim-sulfamethoxazole, streptomycin, fosfomycin, and chloramphenicol resistance genes. A few contained plasmid-mediated quinolone resistance genes. Several samples, especially the respiratory specimens, were also positive for KPC and CTX-M group 1 and 9 genes. Most of the healthy donor specimens were positive for trimethoprim-sulfamethoxazole resistance genes, and many were positive for streptomycin, aminoglycoside, tetracycline, and fosfomycin resistance genes. Interestingly, 39 of the 89 were positive for *npmA*, a relatively recently described pan-aminoglycoside resistance gene (66). These specimens were the only samples positive for this gene. Three specimens contained plasmid-mediated quinolone resistance genes. Fortunately, none were found to contain ESBL or carbapenemase genes. No complex specimens in this study were positive for genes encoding the important carbapenemases OXA-48, VIM, and NDM, and none were positive for the plasmid-mediated colistin resistance gene *mcr-1* (see Table S4 in the supplemental material).

These sets of samples did not appear to contain especially virulent strains of K. pneumoniae. The yersiniabactin siderophore genes were, by far, the most prevalent of the virulence genes tested, although positive samples made up less than half of the K. pneumoniae-positive samples. No specimens were positive for *rmpA*, the regulator of mucoid phenotype gene, including the ST380-containing sample, and few were positive for the salmochelin siderophore genes, which are associated with invasive K. pneumoniae infection (54). One respiratory specimen that contained an ST14 strain was positive for a K2 capsule type by partial *wzi* sequencing. K2 strains of K. pneumoniae are associated with hypermucoviscosity and hypervirulence, as previously mentioned. However, this respiratory sample was not positive for *rmpA*, and a recent study proposed that the presence of multiple siderophore system genes (linked to K1 or K2 capsule genes) explains hypervirulence rather than capsule type (54). In our data, K. pneumoniae-containing samples were positive for multiple siderophores or other virulence-associated genes only 15% of the time. Sequencing of *wzi* revealed a variety of capsule types and cases in which the same clonal groups had different *wzi* genotypes and in which they had the same genotype. This character would help identify or rule out a transmission event when patients carrying the same strain are found.

On an interesting note, in the healthy donor fecal samples collected from members of the same families over time, out of the nine K. pneumoniae-positive samples, only two came from the same person over time. The characterization assays suggest that the same strain of K. pneumoniae was present at both time points. K. pneumoniae-positive samples were found in multiple members of two of the seven families. In one of these families, the positive members carried strains different from one another, and in the other, it appears that two members had CG37 isolates with the same capsule type. The sample set is too small to draw conclusions from these data; however, the data raise interesting questions about community K. pneumoniae carriage.

### Validation of KlebSeq strain identification in specimens.

Table 3 shows MLST results from WGS data of six isolates cultured from specimens run on KlebSeq and from MLST PCR and sequencing of 11 specimens run on KlebSeq. In each case, KlebSeq appears to have identified the correct strain. Two isolates for which no strain type was identified by KlebSeq have novel STs. Sample TG75900 was identified as K. quasipneumoniae by KlebSeq and typed as ST196 on the basis of whole-genome data. This genome was added to the phylogeny of the three species and clustered with K. quasipneumoniae (Fig. 5). MLST of the specimen DNA did not yield results for all of the MLST loci of all 11 samples, which is to be expected given the complexity of the specimen DNA sample. In cases where only partial data were retrieved, at least three alleles from each match the strain identified by KlebSeq.

**TABLE 3:** Results of KlebSeq strain identification validation by MLST of isolates cultured from specimens tested by KlebSeq and MLST of specimens tested by KlebSeq

Sample	Type	KlebSeq identification of original specimen	No. of loci retrieved from sequence data	ST by MLST
TG69923	Isolate	CG29	7	Novel; DLV^*a*^ of ST29
TG75899	Isolate	CG392	7	ST392
TG75900	Isolate	K. quasipneumoniae	7	ST196
TG75901	Isolate	ST133	7	Novel; SLV^*b*^ of ST133
TG75902	Isolate	No strain ID	7	Novel; DLV of ST248
TG75911	Isolate	Mixture of K. pneumoniae with no strain ID and K. variicola	7	Novel; TLV^*c*^ of ST633
TG69737	Urine	CG34	7	Novel; 4 alleles match ST34
TG69766	Urine	CG45	6	6 alleles match ST45
TG69776	Urine	CG111	7	Novel; DLV of ST111
TG69861	Respiratory	No strain ID	7	Novel; SLV of ST393
TG69865	Respiratory	ST134	6	5 alleles match ST134
TG69871	Respiratory	CG37	3	3 alleles match ST37
TG69883	Respiratory	ST258	7	Novel; 3 alleles match ST258
TG73885	Respiratory	CG36	7	Novel; 3 alleles match ST36
TG73911	Respiratory	Mixture of K. pneumoniae with no strain ID and CG36	6	4 alleles match ST36
TG73916	Respiratory	Inner ST14	7	Novel; 4 alleles match ST14
TG74003	Respiratory	Mixture of K. pneumoniae no strain ID and CG36	7	Novel; DLV of ST461

a DLV, double-locus variant.

b SLV, single-locus variant.

c TLV, triple-locus variant.

## DISCUSSION

In the United States, HAI is estimated to affect 1 in 25 hospital patients, totaling hundreds of thousands of patients, with significant mortality (2). HAIs have a significant impact on health care costs; a 2009 CDC report estimated upwards of $45 billion in annual additional cost (67). Infections with AMR organisms cause significantly higher mortality rates, significantly more intensive care unit (ICU) admissions, and significant excess costs, including hospitalization, medical care, and antimicrobial therapy, than do infections with susceptible strains (5, 68). HAI prevention measures, although costly in and of themselves (69), have the potential to save thousands of lives and billions of dollars (67). Periodic patient screening and isolation of AMR organism carriers have proven successful in controlling transmission and outbreaks in several hospitals (31, 34–37). Use of a highly informative screening and surveillance tool such as KlebSeq has cost-effective and life-saving potential.

Early detection of colonization of health care patients by K. pneumoniae, especially multidrug-resistant K. pneumoniae, would allow health care staff to make more informed patient management decisions. In outbreak situations, rapid identification of transmissions before subsequent infections would allow for proactive measures to curb an outbreak. In nonoutbreak situations, identification of particular strains and AMR genes would help to assess the risk of K. pneumoniae carriage to the host patient, as well as to other patients, as some strains are more closely associated with adverse outcomes (e.g., outbreaks, HAI, AMR, and treatment failure) than others (7-13, 70). Although our understanding of many Klebsiella virulence factors is limited, identification of virulence genes gains us understanding of the correlations between particular virulence factors and the risk of disease (54). Additionally, many K. pneumoniae infections, including HAIs and non-multidrug-resistant infections, are caused by nonepidemic, lesser-known strain types (64, 65). Classifying the K. pneumoniae isolate in each patient sample would help an institution to decide when and which intervention procedures should be enacted and also to understand more about transmission dynamics and local strain type circulation.

KlebSeq has several characteristics that make it attractive as a health care screening approach. With a single assay, enough information is garnered about a patient's Klebsiella carriage status to contribute greatly to patient management and to infection control decisions. Indexing samples by means of the universal tail during sample preparation allows the characterization of a large number of specimens in one run, minimizing sequencing costs per specimen and allowing for high-throughput screening of hundreds of patient samples simultaneously at a cost of tens of dollars per patient. KlebSeq uses DNA extracted directly from a specimen, so targets from entire populations of a species are analyzed to capture different strains in the same sample, which can be numerous (71, 72). If culture-based methods are used for screening, different strains are missed when one genotype (i.e., colony) is chosen for characterization, and the resulting information is limited. Additionally, culture-based methods can miss “silent” multidrug-resistant K. pneumoniae strains that test negative for carbapenemases *in vitro* (16), and if used for high-throughput screening, they can be laborious, time-consuming, costly, and subjective (31, 38). If screening of large numbers of patients by amplicon sequencing is cost-prohibitive, it can be limited to the highest-risk groups of patients, i.e., long-term care facility patients (31, 73), travelers returning from regions where Klebsiella carriage is endemic (74, 75), ICU patients (28), patients with previous K. pneumoniae carriage (75–77), patients who have shared a room with a known carrier (78) or case contacts of carriers (79), those who have recently taken antibiotics (80, 81), or patients on mechanical ventilation or enteral feeds or who have had prior Clostridium difficile infections (82). Additionally, using ASAP makes the analysis in KlebSeq streamlined and results are easily interpretable. Lastly, the amplicon sequencing and ASAP package is customizable and updateable (45). Individual assays can be added or removed, adding only the cost of new primers.

The results we present here show that KlebSeq is effective with DNA from numerous sample types, including pure organism culture, or complex, multiorganism samples and swab samples with low-level microbial DNA in a presumably high human DNA background without culture methods. In addition to clinically important clonal lineages of K. pneumoniae, KlebSeq also reliably distinguishes among the Klebsiella species, two of which, K. quasipneumoniae and K. variicola, are continuously misidentified as K. pneumoniae (as exemplified in our study) and cause invasive disease (83, 84). Additionally, we highlight several instances where culture methods failed to produce a positive K. pneumoniae result, including one sample that contained the critical ST258 strain. The sensitivity of KlebSeq is superior to that of culture-based methods for complex specimens, lowering the risk of false negatives in patient screening. We identified dozens of AMR and virulence genes within individual samples, demonstrating the additional function of profiling for clinically important characteristics, and were able to distinguish minor genotype differences that confer different phenotypes, such as the *gyrA* gene, *aac*(*6*′)-*Ib* versus *aac*(*6*′)-*Ib-cr*, and the *wzi* gene.

Notably, our data show that healthy individuals may carry clinically important strains of K. pneumoniae and frequently K. oxytoca, as well as many AMR genes and siderophore virulence systems. For our purposes, these healthy donor fecal DNA samples were used to validate the use of our amplicon sequencing approach with highly complex fecal metagenome samples. Much more study is needed to elucidate the implications of healthy host carriage of known pathogenic strains of K. pneumoniae and their virulence factors. Furthermore, the fact that we observed carriage of the hypervirulence-associated ST380 strain from a healthy person and the hypervirulence-associated K2 capsule type in an ST14 strain from a respiratory infection lends credence to the idea that we need much more information about K. pneumoniae carriage strains to be able to draw conclusions about these associations. Aside from these important observations, other observations from these data raise questions about the dynamics of K. pneumoniae carriage and microbiome sharing. The fact that there were not more cases of positive results from the same person at multiple rather than single time points is interesting. This could be due to intermittent shedding of K. pneumoniae in feces, intermittent colonization by K. pneumoniae, or heterogeneity in the sample itself, underrepresenting the full microbial community when a small sample is taken; this observation further warrants periodic rather than one-time screening of patients at risk. Of the two instances where two family members carried K. pneumoniae, each tells a different story about microbiome sharing. An amplicon sequencing-based diagnostic approach would facilitate longitudinal patient screening because of ease of use and limited costs.

Among the many pathogens encountered in health care institutions, we focus only on Klebsiella because of its high priority in public health and the high risk of CPE establishment in a facility. Additionally, although many other HAI agents cause devastating and costly infections, directing a complex assay such as KlebSeq at a subset of those agents greatly simplifies the validation process and speeds the availability of assay results (especially in the case of validation for FDA approval [85]). KlebSeq is an important step toward a comprehensive yet accessible tool for all pathogen identification and characterization. Metagenomic analysis is attractive in its breadth of coverage capabilities, but its current costs and complexities are prohibitive. Amplicon assays targeting the other ESKAPE (Enterococcus faecium, Staphylococcus aureus, Klebsiella pneumoniae, Acinetobacter baumannii, Pseudomonas aeruginosa, and Enterobacter species) pathogens and more AMR determinants could relatively easily be added to the tool described here, with a step-by-step validation process. We have not found the limit of the multiplexing capacity. (We run three multiplexed PCRs merely because the PCR volume limits the number of primers that can be added; future assays will utilize primers at a higher concentration to test the multiplexing limit.) The multiplexing limit is also presumably dependent on the number and sizes of targets that are present in a sample, potentially exhausting the polymerase or sequencing space. These pieces are unpredictable for specimens of unknown content. Lastly, our report serves as evidence in favor of the concept that highly multiplexed amplicon sequencing is one good answer to the call for early detection tools in infection control.

Overall, the KlebSeq method was able to accurately and consistently identify and characterize Klebsiella from complex specimens. A limitation of our study is that clonal group identification in the complex specimens was not confirmed by isolation and WGS of the Klebsiella isolate from the specimen. Additionally, profiling of complex specimens directly for AMR and virulence genes, most of which are on mobile elements, can be confounding, as it cannot be known which organism carries the genes of interest. Third, our validation efforts focused on the detection of targets but did not characterize the limits of that detection, and we saw evidence that KlebSeq may not find all of the variants of all of the AMR genes. However, KlebSeq is designed for screening and periodic surveillance in high-risk situations with a rule-in/rule-out determination of the possibility of transmission events and through identification of high-risk multidrug-resistant or epidemic strains of Klebsiella. For these purposes, KlebSeq is ideal. The specimen types used for validation could be considered a limitation, as we did not test rectal swabs, a specimen type commonly used for CPE surveillance. However, we show that KlebSeq works with different swab types and fecal specimens, which addresses the challenges of detection in rectal swabs. The turnaround time from sample collection to result is dependent only on current technology (not organism culture), and we recently conducted a proof-of-concept study of a 24-h sample-to-answer test with different targets (data not shown). This test was done on an Illumina MiSeq with only 60 cycles. Other platforms and upcoming technology may allow this turnaround time to be decreased even further.

Rapid amplicon sequencing with automated analysis and reporting is a promising response to the need for constant surveillance for highly transmissible or highly drug-resistant pathogens. Our model system, directed at Klebsiella, can easily be adapted to multiple other pathogens and to different purposes, such as environmental sampling and community host screening and, as smaller, more on-demand next-generation systems become available, to diagnostics and individual patient monitoring. The targeted, highly multiplexed nature of amplicon sequencing and the ability to interpret the data instantly make it an applicable tool for health care facility surveillance. As these technologies are adopted, considerable coordination within the health care facility is paramount to the success of infection and outbreak prevention, with the integration of isolation and barrier precautions, excellent communication, and good stewardship. Nevertheless, several institutions have shown that the combination of surveillance and systematic response reduces outbreaks and multidrug-resistant infections (31, 33–37).

## Supplementary Material

Supplemental material
