# Supplementary material for: KlebSeq, a Diagnostic Tool for Surveillance, Detection, and Monitoring of Klebsiella pneumoniae
Source: J Clin Microbiol. 2016 Sep 23;54(10):2582–96. doi: 10.1128/JCM.00927-16 (PMC5035412; doi:10.1128/JCM.00927-16)
Supplement: Supplemental material [file supp_54_10_2582__index.html]

Supplemental material 

# KlebSeq, a Diagnostic Tool for Surveillance, Detection, and Monitoring of Klebsiella pneumoniae

## Supplemental material

- Supplemental file 1 -

  Tables S1 (Accession numbers of samples used for phylogenetic analysis), S2 (Samples used for KlebSeq validation), S3 (KlebSeq assay information), and S4 (KlebSeq results on specimens)

  XLSX, 230K
